# A New Method for Assessing How Sensitivity and Specificity of Linkage Studies Affects Estimation

**DOI:** 10.1371/journal.pone.0103690

**Published:** 2014-07-28

**Authors:** Cecilia L. Moore, Janaki Amin, Heather F. Gidding, Matthew G. Law

**Affiliations:** 1 The Kirby Institute, UNSW Australia, Sydney, New South Wales, Australia; 2 School of Public Health & Community Medicine, UNSW Australia, Sydney, New South Wales, Australia; Brighton and Sussex Medical School, United Kingdom

## Abstract

**Background:**

While the importance of record linkage is widely recognised, few studies have attempted to quantify how linkage errors may have impacted on their own findings and outcomes. Even where authors of linkage studies have attempted to estimate sensitivity and specificity based on subjects with known status, the effects of false negatives and positives on event rates and estimates of effect are not often described.

**Methods:**

We present quantification of the effect of sensitivity and specificity of the linkage process on event rates and incidence, as well as the resultant effect on relative risks. Formulae to estimate the true number of events and estimated relative risk adjusted for given linkage sensitivity and specificity are then derived and applied to data from a prisoner mortality study. The implications of false positive and false negative matches are also discussed.

**Discussion:**

Comparisons of the effect of sensitivity and specificity on incidence and relative risks indicate that it is more important for linkages to be highly specific than sensitive, particularly if true incidence rates are low. We would recommend that, where possible, some quantitative estimates of the sensitivity and specificity of the linkage process be performed, allowing the effect of these quantities on observed results to be assessed.

## Introduction

Record linkage is the task of bringing together information from two or more different sources that pertain to the same individual. Increasingly it is used to determine outcomes, particularly cancer and mortality, in large cohort studies or registry based populations [1]–[10]. There are a number of advantages of record linkage studies. The ability to use existing administration data can significantly increase cost-efficiency. In addition, the size and representativeness of the study sample may be increased [11]. However, record linkage studies are constrained by the quality of the datasets being linked and by the methods of linkage used [11]–[13]. Particularly, linkage can be more complex if the amount or quality of identifying data for individuals are limited. In these cases, probabilistic linkage methods have become widely used [14].

Probabilistic linkage assigns weights to potentially matched records, based on the contribution from each partial identifier [14], [15]. Being more unique, agreement on first and last name in both records, for example, will contribute more weight than agreement on sex. To maximise accuracy, several matching strategies and subsequent clerical review are usually employed [14], [15]. While there are an increasing number of studies aimed at the development and improvement of record linkage procedures, no probabilistic linkage is perfect. Some records that are true matches will fail to be linked and other truly non-matching records will be incorrectly linked. While the objective of any linkage strategy will be to maximise sensitivity and specificity, thereby minimising misclassification of outcomes, a certain degree of error will remain. Furthermore the trade-off between sensitivity/specificity means any improvement in sensitivity, must be at some cost in terms of poorer specificity and the increased likelihood that false links will be made.

It has long been recognised that misclassification, and as a result linkage errors, can lead to biased results [16]. While most researchers are aware that poor sensitivity will result in under estimation of event rates and poor specificity will result in false positive matches and an over estimation of event rates, few researchers have attempted to quantify how this may have impacted on their study’s findings and outcomes. Even where authors of linkage studies have attempted to estimate sensitivity and specificity based on subjects with known status or estimates of sensitivity and specificity are provided by the data linkage unit, the effects of false negatives and positives on event rates are not often described [17]–[19].

The purpose of this paper is to provide further assistance to researchers who are attempting to appraise the possible impact of errors in the linkage process. The problem of missing linkage is viewed as misclassification of outcome and with this in mind we present a simple quantification of the effect of sensitivity and specificity on incidence and event rates as well as the resultant effect on relative risks. Furthermore, we present a derived formula that allows the “true” number of events in a linkage study to be estimated from the observed number of events based on the sensitivity and specificity of the data linkage and illustrate its use in a linkage study [17].

## Methods

### Relationship between sensitivity, specificity and estimates of incidence

Table 1 outlines the relationship between true and observed event data. The *sensitivity* (SE) is the probability of detecting an event via linkage if an event has truly occurred and is equal to TP/(TP+FN). The *specificity* (SP) is the probability of not detecting an event if the event is truly absent and is equal to TN/(TN+FP). N is the number of individuals in the population and equals TP+FP+TN +FN. 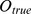 is the actual number of events in the population which equals TP+FN and 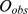 is the observed number of events and equals TP+FP. Thus the following formula (1) can be used to ascertain the observed number of events by weighting the number of true events by the sensitivity and the number of non-events by the specificity.
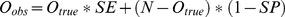
(1)


**Table 1:** Distribution of study events according to both actual event and observed event (by linkage).

		True event		
		Yes	No	
**Observed event (by linkage)**	**Yes**	***TP*** * (true positives)*	***FP*** * (false positives)*	***TP+FP***
	**No**	***FN*** * (false negatives)*	***TN*** * (true negatives)*	***FN+TN***
		***TP+FN***	***FP+TN***	

By substituting the nomenclature above into the right hand side of formula one,
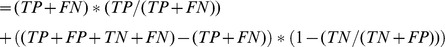



Cancelling the TP+FN in the first part of the equation and simplifying throughout results in
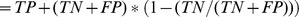



Multiplying the TN+FP the last part of the equation results in


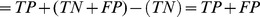
, which is our definition of observed number of events (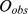).

**Figure 1:**
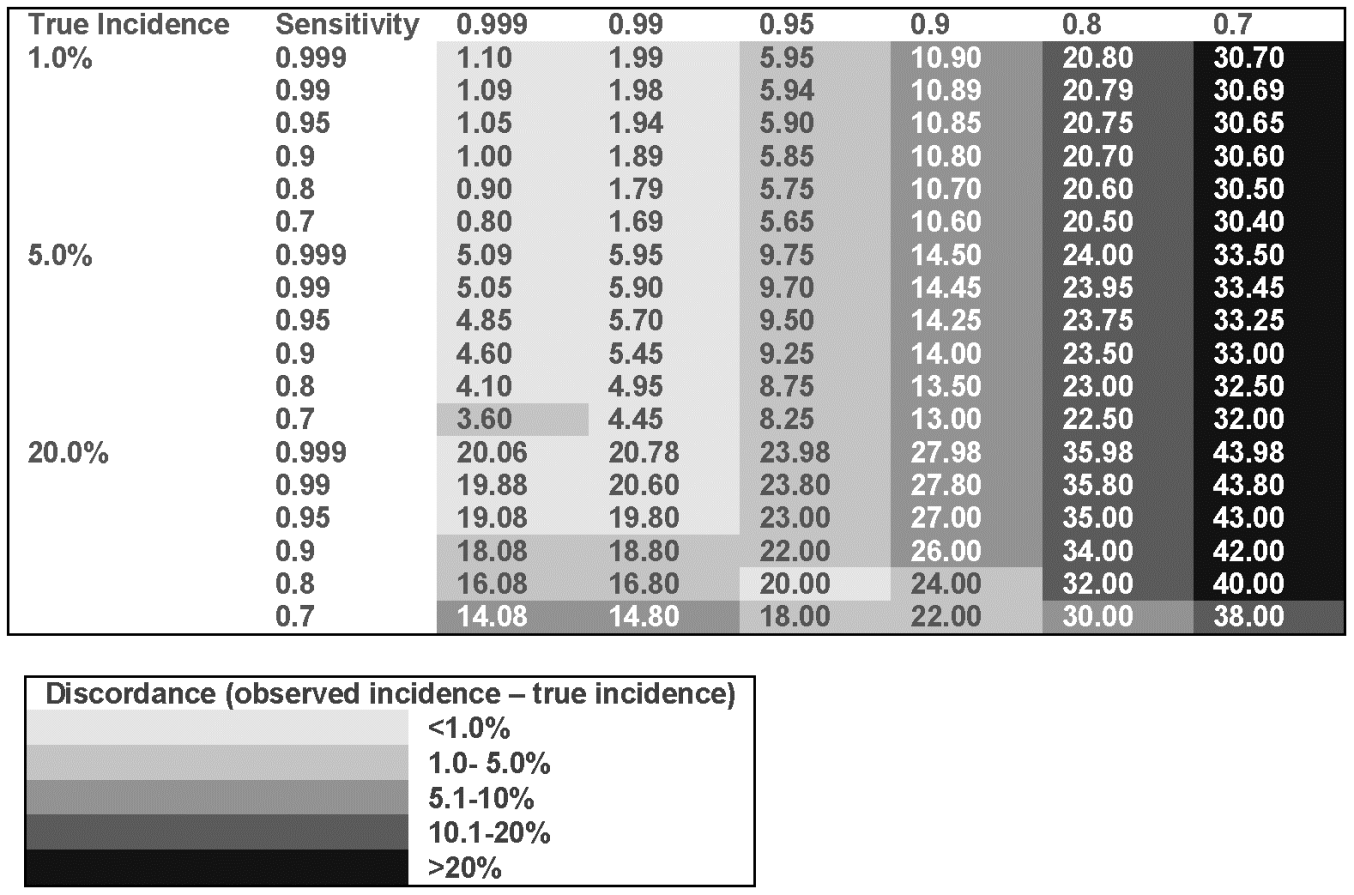
The effect of changing sensitivity and specificity on observed incidence when true incidence is 1.0%, 5.0% and 20.0%.

Misclassification as a result of poor sensitivity results in an under estimation of the number of events, while poor specificity results in an over estimation of events. Figure 1 demonstrates that for a range of true incidence rates, the observed incidence is more biased by poor specificity than by poor sensitivity. Furthermore, it illustrates that regardless of the sensitivity, if specificity is high then the true event rate is always under estimated. Thus if any bias occurs, it would be towards the null, allowing study results to be interpreted more robustly.

### Relationship between sensitivity, specificity and estimates of relative risk

Linkage studies are often used to examine the effect of certain risk factors on a specific event. The impact of sensitivity and specificity on incidence carries through to the estimation of effect size such as relative risks (RR) or standardised ratios and can result in significant bias in these estimators. This effect is illustrated by applying equation 1 to determine the observed number of events in the population exposed to the risk factor, separately to those not exposed to the risk factor. For example, consider a population of 10,000, half of whom are exposed and the other half not exposed. If we set the true event rate to be 10% in the exposed and 5% in the non-exposed populations, the relative risk is given by
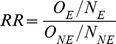
(2)Where *N_E_* is the number of individuals in the exposed population, *O_E_* is the number of events in the exposed population, *N_NE_* is the number of individuals in the non-exposed population and *O_NE_* is the number of events in the non-exposed population.

So the true relative risk is given by:



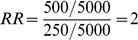



However if the number of events can only be determined with a sensitivity of 0.90 and specificity of 0.95 then by applying equation 1: the observed events in the exposed population become:
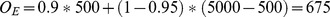
the observed events in the non-exposed population become:



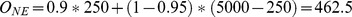



And hence 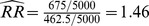.

The observed RR 1.46 is a considerable under estimate of the true RR = 2.

**Figure 2:**
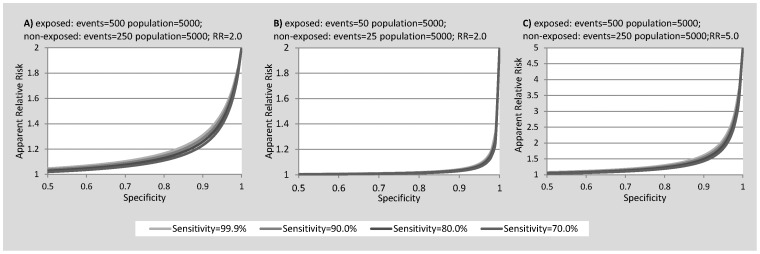
(A) Effect of sensitivity and specificity on estimated relative risk (RR); and effect on this relationship at lower incidence (B) and higher true relative risk (C).

Figure 2 depicts the estimated relative risks derived by including various combinations of sensitivity and specificity in equation 1 in situations of both lower incidence (B) and higher true relative risk (C). It can be seen across all scenarios that the derived RR is an under estimate of the true RR, with bias towards the null. Again, changes in specificity have a greater impact than changes in sensitivity. The greatest impact of poor specificity is seen in scenarios where incidence rates are low or the relative risk is high. Figure 2B depicts the scenario where incidence is reduced to 1% in the exposed. In this instance, even when specificity is 0.999 and sensitivity is 0.99, the estimated relative risk is 1.83, substantially lower than the estimated RR of 1.98 when incidence is 10% in the exposed (Figure 2A). Figure 2C depicts the scenario where the true RR is 5. In this instance, the estimate of RR decreases from 4.81 to 3.65 (24.1%) when sensitivity is held at 0.99 and specificity reduced from 0.999 to 0.99. By comparison, when the true RR is 2 the estimated RR decreases from 1.98 to 1.83 (7.6%) (Figure 2A).

Additionally, if the specificity of event incidence is 100%, then the relative risk will not be biased irrespective of sensitivity. To examine this further, consider our application of equation 1 to determine event rates in exposed and non-exposed individuals.
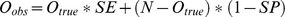
If specificity is 100%, the second half of the equation 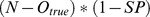 becomes zero and can be eliminated. Therefore the observed relative risk would be



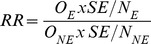



Sensitivity terms will cancel in the equation and thus the equation for observed relative risk simplifies to the equation for the true relative risk
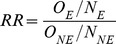



### Adjusting observed events

So far, we have described the effect of sensitivity and specificity on the observed number of events. However, it is more common in linkage studies that the observed number of events is known and the aim is to determine how much the observed number of events is biased from the true number of events. If the sensitivity and specificity for a linkage is known, the true number of events, 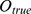, can be derived by adjusting the number of observed events, 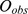, using equation 1 and Table 1 as follows.

The observed number of events is described as:
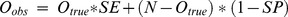
(3)




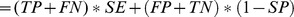



The observed number of non-events is described as:
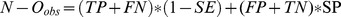
(4)


Multiplying equation (3) by specificity and equation (4) by 1 minus specificity gives
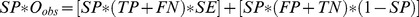
(5)




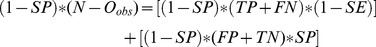
(6)


Solving these two simultaneous equations for the true number of events, 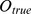, gives
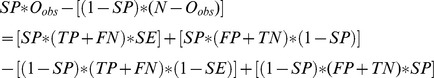




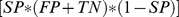
 cancels from the right hand side of the equation to get
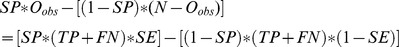



Rearrange the right hand side of the equation to get:
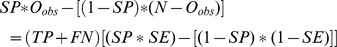



Simplifying the left hand side of the equation to get:
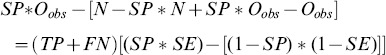





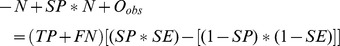



Multiplying both sides of the equation by 
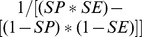
 to get:
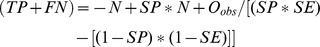



Simplifying this equation you get:
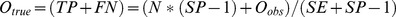
(7)


If linkage study estimates of sensitivity and specificity are available, then these estimates can be used with equation 7 to assess the effect of false positive and negative matches due to the linkage process on overall observed results. Further, the adjusted number of events can then be used to determine adjusted measures of effect.

## Results

### Illustration using the NSW prison inmate linkage study

Adjusted measures of effect can be derived from linkage studies which are able to estimate sensitivity and specificity either via internal or external validation. This is illustrated by an empirical example from the population-based studies of prisoners in New South Wales [20]. A total of 85,203 inmates incarcerated in the NSW prison system between January 1988 and December 2002, were linked with records in the National Death Index database from January 1988 to December 2002. Linkage was performed using the probabilistic record linkage software package Integrity using the personal identifiers: full name, date of birth, sex and date of last contact with the prison system. The validity of the data linkage was assessed in a sub-study in which outcomes in 7,869 prisoners of known vital status at the end of the study period were compared to that determined by probabilistic linkage [17]. A total of 311 prisoners died while in prison and so were known to be dead. A total of 7,558 were alive in prison at the end of the study period and so were known to be alive. Data for these prisoners with known vital status were linked with the National Death Index using the same method used in the full study, with the results given in Table 2.

**Table 2:** Distribution of prisoner vital status on the basis of record linkage and known vital status.

		Records with known vital status		
		Dead	Alive	
**Vital status according to data linkage**	**Dead**	275	23	298
	**Alive**	36	7535	7571
		**311**	**7558**	

The sensitivity and specificity of linkage was estimated to be 88.4% and 99.7% respectively. For the study cohort, a total of 5,137 of 85,203 inmates were found to have died according to data linkage. This compared to an expected 1,323 events, giving an SMR (standardised mortality ratio) of 3.9. Equation 7 above was used to determine the true number of events, in this case deaths, and then applied to the SMR as follows:
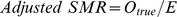
where 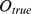 is the number of deaths determined by data linkage adjusted for sensitivity and specificity of the linkage and *E* is the expected number of deaths based on mortality rates in the comparator population.

After adjusting for the sensitivity and specificity obtained in the sub-study using equation 7, the adjusted number of deaths was 5,540 to give an adjusted SMR for mortality of 4.2. In this case, where the linkage process was shown to have good sensitivity and very high specificity, the effect of false positive and negative matches is estimated to have biased the study SMR slightly towards the null.

The relative risk can be similarly adjusted. Among male prisoners, the RR for death in those with psychiatric hospital admission was determined. In those admitted to psychiatric hospitals and not admitted, respectively, the following were reported: observed deaths (467, 4247), expected deaths (85.25, 1183.43) and populations (3919, 72444) to give a RR of 2.03. After applying these data to equation 7 to determine the number of true deaths in each group adjusted for the reported sensitivity (88.4%) and specificity (99.7%), the true relative risk for death following psychiatric hospital admission is 2.09. This result again shows that misclassification in the linkage resulted in a slight bias towards the null.

## Discussion

Results of epidemiological studies using outcomes determined by linked datasets are affected by errors in linkage. To date, methods to quantitatively assess the effect of misclassification on observed study events in linkage studies have not been described. This study develops and tests a simple formula for adjusting observed events and relative risk by known estimates of sensitivity and specificity. This formula and the conceptual framework behind it are analogous to the methods used to adjust for misclassification when calculating odds ratios and hazard ratios in general epidemiological studies [21].

If the estimates of sensitivity and specificity are not valid, then it is possible for the formula to give nonsense adjusted estimates, for example, negative values if specificity is estimated to be low. The formula is also only a simple, approximate adjustment. It only adjusts the observed number of events, and does not adjust estimates of person-years at risk that would also be affected. However, for events that are relatively uncommon, the person-years at risk would be altered only minimally and are probably not an important component of uncertainty.

A further limitation is that our method does not allow for uncertainty in the estimates of sensitivity and specificity. We would recommend that the formula is used as a quantitative assessment of sensitivity and specificity of the linkage process, but that unadjusted results are presented as the main study findings. Our analyses have assumed that errors in linkage are random or non-differential and demonstrate how random error will bias outcomes towards null findings. A review by Bohensky *et al.* reminds us that there are many non-random factors which impact on linkage producing a range of less unpredictable biases [22].

Our results have important implications for study design. Comparisons of the effect of sensitivity and specificity on incidence indicate that it is much more important for linkages to be highly specific than sensitive, particularly if true incidence rates are low. Typically, linkage studies commonly use probabilistic linkage methods and these methods are primarily designed to improve the sensitivity of the linkage process. However, the trade-off between sensitivity and specificity means any improvement in sensitivity must be at some cost in terms of poorer specificity. Despite this trade-off, our results suggest that linkage methods that maximise specificity will lead to the most robust study results, particularly for events that are rare. Other approaches which have accounted for the impact of linkage error on statistical inference include work done by Scheuren and Winkler [23] Scheuren and Winkler [24], and Lahiri and Larsen [25]. They considered linear regression methods that correct for linkage error by applying a bias correction estimated from linkage weights to the ordinary least squares (OLS) estimate. These methods are useful when it is not possible to validate the linkage against a gold-standard sub-sample.

As a result of our deliberations we would recommend that analyses which only consider exact linkage matches, an approach that would probably result in close to 100% specificity but at a possibly much lower sensitivity, should routinely be included as sensitivity analyses. Furthermore, in all linkage studies we would recommend that some quantitative estimates of the sensitivity and specificity of the linkage process be performed if possible, allowing the effect of these quantities on observed results to be assessed.
